# MicroRNA-134 as a potential plasma biomarker for the diagnosis of acute pulmonary embolism

**DOI:** 10.1186/1479-5876-9-159

**Published:** 2011-09-24

**Authors:** Junjie Xiao, Zhi-Cheng Jing, Patrick T Ellinor, Dandan Liang, Hong Zhang, Ying Liu, Xiaoli Chen, Lei Pan, Robert Lyon, Yi Liu, Lu-Ying Peng, Xingqun Liang, Yunfu Sun, Laurentiu M Popescu, Gianluigi Condorelli, Yi-Han Chen

**Affiliations:** 1 Key Laboratory of Arrhythmias, Ministry of Education, China (East Hospital, Tongji University School of Medicine), Shanghai, China; 2 Institute of Medical Genetics, Tongji University, Shanghai, China; 3 Department of Pulmonary Circulation, Shanghai Pulmonary Hospital, Tongji University, Shanghai, China; 4 Cardiovascular Research Center and Cardiac Arrhythmia Service, Massachusetts General Hospital, Boston, USA; 5 Department of Medicine, University of California San Diego, La Jolla, USA; 6 Department of Cellular and Molecular Medicine, Carol Davila University of Medicine and Pharmacy, Bucharest, Romania

## Abstract

**Background:**

Acute pulmonary embolism (APE) remains a diagnostic challenge due to a variable clinical presentation and the lack of a reliable screening tool. MicroRNAs (miRNAs) regulate gene expression in a wide range of pathophysiologic processes. Circulating miRNAs are emerging biomarkers in heart failure, type 2 diabetes and other disease states; however, using plasma miRNAs as biomarkers for the diagnosis of APE is still unknown.

**Methods:**

Thirty-two APE patients, 32 healthy controls, and 22 non-APE patients (reported dyspnea, chest pain, or cough) were enrolled in this study. The TaqMan miRNA microarray was used to identify dysregulated miRNAs in the plasma of APE patients. The TaqMan-based miRNA quantitative real-time reverse transcription polymerase chain reactions were used to validate the dysregulated miRNAs. The receiver-operator characteristic (ROC) curve analysis was conducted to evaluate the diagnostic accuracy of the miRNA identified as the candidate biomarker.

**Results:**

Plasma miRNA-134 (miR-134) level was significantly higher in the APE patients than in the healthy controls or non-APE patients. The ROC curve showed that plasma miR-134 was a specific diagnostic predictor of APE with an area under the curve of 0.833 (95% confidence interval, 0.737 to 0.929; P < 0.001).

**Conclusions:**

Our findings indicated that plasma miR-134 could be an important biomarker for the diagnosis of APE. Because of this finding, large-scale investigations are urgently needed to pave the way from basic research to clinical utilization.

## Background

Acute pulmonary embolism (APE) is a common cardiovascular emergency associated with a substantial morbidity and mortality [1-3]. APE accounts for 50, 000 to 100, 000 deaths a year in the United States alone and approximately 10% of deaths in European hospitals [2,4]. The annual incidence of APE in the United States and Europe is approximately 100 cases per 100, 000 individuals [2,4]. Despite the morbidity associated with APE, the diagnosis is frequently missed due to the variable clinical presentation [5,6].

Diagnostic testing for APE has been extensively studied and ranges from biomarkers, such as the D-dimer assay, to radiologic imaging, including CT angiography, venous ultrasonography, and ultimately the gold standard of pulmonary venous angiography. Although widely used as a screening tool, D-dimer assays are sensitive but not specific for detecting APE [7]. Novel biomarkers with enhanced diagnostic accuracy would greatly facilitate the diagnosis of APE [3].

In recent years, microRNAs (miRNAs) have been found to play crucial roles in many cellular processes, such as development, proliferation, differentiation and apoptosis. MiRNAs are small, endogenous, single-stranded, noncoding RNAs that regulate gene expression by hybridizing to messenger RNAs (mRNAs) and inhibiting mRNA translation or promoting mRNA degradation [8-11]. The expression pattern of many miRNAs is reflective of various pathophysiologic processes and underlies a large number of diseases [8,12-14]. Circulating miRNAs are also emerging as biomarkers in various diseases, including cancer, drug-induced liver injury, heart failure, type 2 diabetes, stable angina pectoris, and acute coronary artery syndromes [8,12-23]. We therefore sought to explore plasma miRNAs as biomarkers for the diagnosis of APE.

## Methods

### Patient populations

Between February 2010 and July 2010, patients with a high clinical probability of APE or those with a low/intermediate probability and a positive D-dimer enzyme-linked immunosorbent assay test (> 500 μg/L) in the Shanghai Pulmonary Hospital, Tongji University underwent testing to confirm APE. In accordance with existing guidelines, these patients underwent a pulmonary angiography, contrast-enhanced computed tomography (CT) pulmonary angiography or a ventilation-perfusion lung scan, alone or in combination [24]. After the diagnosis of APE, transthoracic echocardiography was performed to detect (or exclude) right ventricular dysfunction (i.e., dilation of the right ventricle with an end-diastolic diameter > 30 mm from the parasternal view, or the right ventricle appearing larger than the left ventricle from the subcostal or apical view) combined with right atrial hypertension (absence of inspiratory collapse of the inferior vena cava) in the absence of left ventricular or mitral valve disease. Serum cardiac troponin I and BNP were used as markers of myocardial injury or right ventricular dysfunction, respectively. Afterwards, risk stratification was conducted according to the guidelines [24]. Briefly, high-risk APE is diagnosed if shock or hypotension occurs. Intermediate-risk APE is confirmed when at least one right ventricular dysfunction or one myocardial injury marker is positive. Low-risk APE is confirmed when all right ventricular dysfunction and myocardial injury markers are negative [24]. During this period, 35 patients were confirmed to have an APE, and the 32 patients that gave informed consent were enrolled in this study. The controls were 32 healthy volunteers. Twenty-two patients (7 with pneumonia, 7 with unstable angina pectoris, 3 with acute myocardial infarctions, 2 with lung cancer, 1 with pleurisy, 1 with bronchiectasis and 1 with asthma) who had reported dyspnea, chest pain, or cough were consecutively recruited as the non-APE cases. Approval was obtained from the ethical committees of the Tongji University School of Medicine. All participants gave written informed consent before enrollment in the study.

### Plasma sampling and RNA isolation

At presentation, blood samples for miRNA detection were collected in EDTA-K2 tubes and processed within 1 hour of collection. After a two-step centrifugation (4°C at 820 × *g *for 10 min, then 4°C at 16000 × *g *for 10 min), the supernatant was transferred to RNase/DNase-free tubes and stored at -80°C.

The total RNA was isolated from the plasma using a mirVana PARIS isolation kit (Ambion, Austin, Texas) according to the manufacturer's instructions for plasma samples. Briefly, 400 μL of human plasma was used to extract the total RNA. Each sample was eluted in 100 μL of RNAse-free water. Spectrophotometric RNA quantification of human plasma samples was not reliable because of undetermined contaminants with an absorbance peak at 270 nm. However, the quantitative real-time reverse transcription polymerase chain reaction (qRT-PCR) was not affected [12]. Thus, all RNA samples were analyzed for miR-16 expression, a stable endogenous reference miRNA, to assess an approximate yield of RNA extraction and to ensure that comparable amounts of starting material were used in each reverse transcription reaction [25-29].

### miRNA expression profile and validation

miRNAs were reverse-transcribed using the Megaplex Primer Pools (Human Pools A v2.1 and B v2.0), and the products were pre-amplified according to the Applied Biosystems' pre-amplification protocol. For the initial screening, Human TaqMan miRNA microarrays (CardA v2.1 and CardB v2.0, Applied Biosystems) covering 667 small, noncoding RNAs were performed on RNA from the plasma of 10 randomly selected APE patients and 10 randomly selected healthy controls. All steps were performed using a 7900HT Fast Real-Time PCR System (Applied Biosystems, Foster City, CA); the results were expressed as Cts and normalized to the calculated median Ct of each sample (ΔCt). The relative expression was calculated using the comparative Ct method (2^-ΔΔCt^). The minimal microarray experiment compliant data were exported to the Gene Expression Omnibus (Platform ID GSE24149).

To minimize the number of false positives, only miRNAs that differed from the healthy controls by more than 10-fold were considered for subsequent validation by qRT-PCR. Single miRNA expression was determined using TaqMan-based miRNA qRT-PCR (Applied Biosystems, Foster City, CA) according to the manufacturer's instructions. Briefly, the 15-μL RT reaction master mix was created with 5 μL of the total RNA sample isolated as described above. qRT-PCR was carried out using the 7900HT Fast Real-Time PCR System on 20 μL of PCR master mix containing 10 μL of TaqMan 2× Universal PCR Master Mix, 1 μL of TaqMan assay, 4 μL of RT products and 5 μL of ddH_2_O. The qRT-PCR reactions were performed in triplicate, and the signal was collected at the end of every cycle. Due to a lack of generally accepted standards, all qPCR data on single miRNA expression were analyzed as unadjusted Ct values and standardized to miR-16, which fulfilled the following criteria: detectable in all samples, low dispersion of expression levels and a null association with APE. To validate miR-16 as a stable internal reference, its stability during extraction was compared to that of synthetic cel-miR-39, an miRNA of *C. elegans *that is not present in humans. Twenty-five fmol of synthetic cel-miR-39 were spiked in after adding the Denaturing Solution (provided in the mirVana PARIS isolation kit) to the human plasma samples to avoid degradation by endogenous RNases, and the RNA was extracted. We measured the expression of cel-miR-39, miR-16 and miR-134 in 10 APE patients and 10 healthy controls selected at random. Afterwards, the expression of miR-134 in APE patients and healthy controls was compared with miR-16 and cel-miR-39 normalizers, respectively.

### Statistical analysis

Data characterized by a normal distribution were expressed as the mean and standard deviation. Relative gene expression is widely presented using the 2^-ΔΔCt ^method [30]. Relative gene expression involves the gene of interest data (Ct_gene of interest_) relative to an internal control gene (Ct_internal control gene_), named delta Ct. The calculated delta Ct ± SD for the different groups (APE and non-APE) were compared with the delta Ct ± SD (SD stands for the standard deviation of the average delta Ct of the group) for the healthy control group and tested for statistical significance. An independent samples *t*-test, Chi-squared test or one-way analysis of variance (one-way ANOVA) was conducted when appropriate. If a significant difference was found, a Bonferroni post-hoc test or Tamhane's T2 post-hoc analysis was conducted to determine which groups differed significantly according to the equal variance criterion. The receiver-operator characteristic (ROC) curve analysis was performed with plasma miRNA distinguishing between APE patients and healthy controls or non-APE patients. The area under the ROC curve (AUC) was estimated to assess the diagnostic accuracy of the miRNA identified. All analyses were performed using SPSS 13.0, and all statistical tests were two-sided. For all analyses, p-values < 0.05 were considered statistically significant.

## Results

### Expression profiles of miRNAs in the plasma of APE patients

Thirty differentially expressed plasma miRNAs were identified in APE patients from healthy controls (Table 1). From these, microRNA-134 (miR-134) and microRNA-410 (miR-410) were selected for further validation based on the changes (> 10-fold) and probability values (P < 0.05).

**Table 1:** Alterations in plasma microRNA levels in patients with an acute pulmonary embolism compared to healthy controls

microRNA	Regulation	Fold Change	p-value
hsa-miR-134	up	25.392	0.047
hsa-miR-410	up	15.221	0.028
hsa-miR-520g	up	6.768	0.048
hsa-miR-485-3p	up	6.170	0.018
hsa-miR-362-5p	up	5.987	0.047
hsa-miR-382	up	5.501	0.042
hsa-miR-548b-5p	up	5.473	0.039
hsa-miR-139-3p	up	5.049	0.024
hsa-miR-197	up	4.363	0.015
hsa-miR-574-3p	up	4.303	0.024
hsa-miR-190	up	4.031	0.039
hsa-miR-489	up	3.656	0.048
hsa-miR-146a	up	3.593	0.030
hsa-miR-320	up	2.470	0.033
hsa-miR-133a	up	2.202	0.042
hsa-miR-20a*	down	5.924	0.005
hsa-miR-7	down	4.990	0.010
hsa-miR-340*	down	4.810	0.039
hsa-miR-923	down	4.255	0.036
hsa-let-7b	down	4.068	0.000
hsa-let-7g	down	3.512	0.009
hsa-miR-768-3p	down	3.166	0.036
hsa-miR-30e	down	3.010	0.011
hsa-miR-610	down	2.852	0.045
hsa-miR-126*	down	2.799	0.006
hsa-miR-301a	down	2.740	0.007
hsa-miR-30d	down	2.720	0.004
hsa-miR-30a	down	2.212	0.003
hsa-miR-374a	down	2.185	0.012
hsa-miR-632	down	1.706	0.043

### Validation of candidate miRNAs

**Figure 1:**
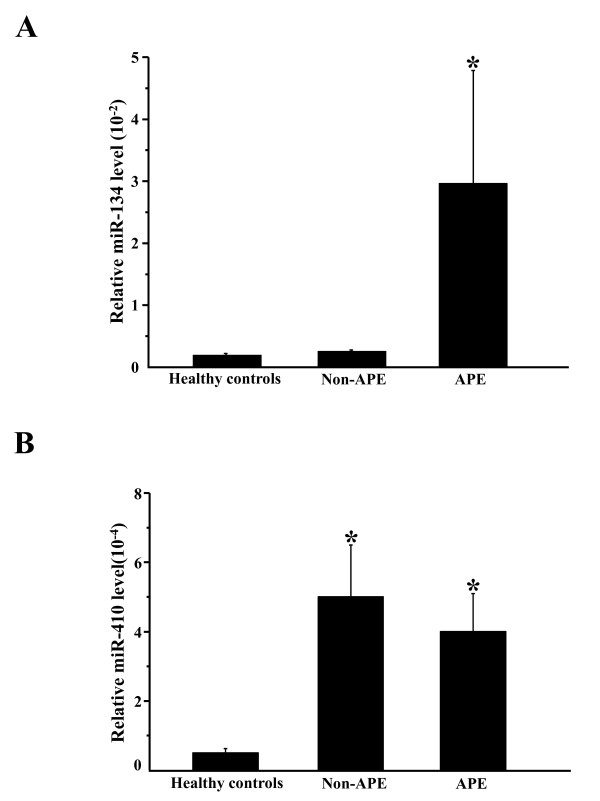
**Relative plasma miR-134 and miR-410 expression levels**. (A) and (B) The relative plasma miR-134 (A) and miR-410 (B) expression level in healthy controls, non-acute pulmonary embolism (non-APE) patients, and APE patients. The expression levels of miR-16 normalize the data. *, P < 0.05, comparison of healthy controls and non-APE patients.

**Figure 2:**
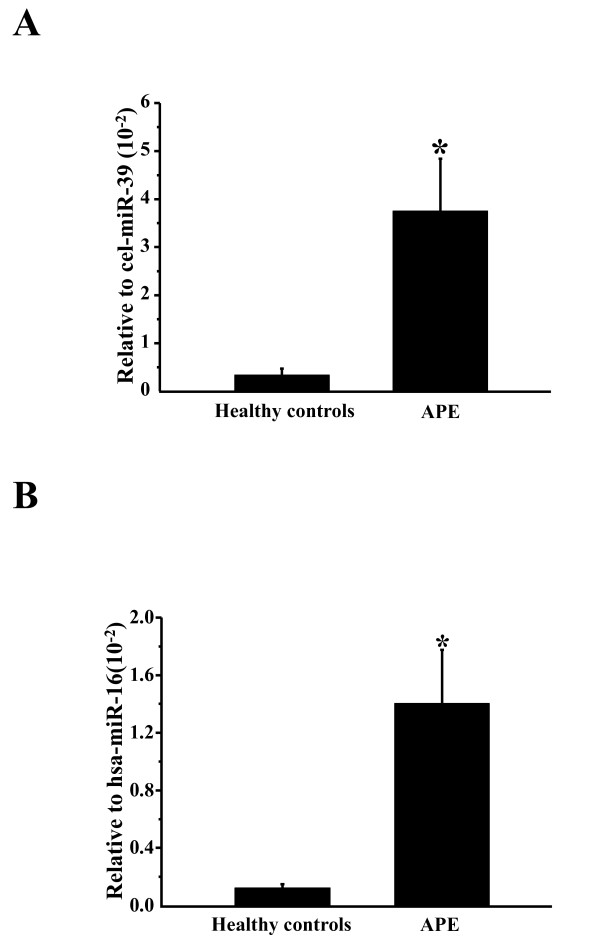
**Relative plasma miR-134 expression levels normalized by cel- miR-39 and hsa-miR-16**. (A) and (B) The relative plasma miR-134 expression levels normalized by cel-miR-39 (A) and hsa-miR-16 (B) in healthy controls and acute pulmonary embolism patients. *, P < 0.05, comparison with healthy controls.

The elevation of miR-134 and miR-410 was validated using TaqMan miRNA qRT-PCR in all participants (Figure 1A-B). The basic clinical characteristics of APE patients and non-APE patients are shown in Table 2. There were no significant differences in gender composition and age between the APE and non-APE groups. The change in candidate miRNAs for the APE group or non-APE group versus the healthy controls is shown in Figure 1. These data have been normalized by the expression levels of miR-16, a widely used endogenous reference miRNA that was also confirmed to be unchanged in our microarrays. In addition, compared to cel-miR-39, miR-16 is stable (Additional file 1 Figure S1). Moreover, we compared the difference in miR-134 expression between 10 APE patients and 10 healthy controls and obtained the same differences in miR-134 expression regardless of whether cel-miR-39 or miR-16 were used as the normalizer (Figure 2), which further supports that miR-16 is a stable reference in this study.

**Table 2:** Clinical characteristics of acute pulmonary embolism (APE) patients and non-APE patients

Characteristics	APE (n = 32)	Non-APE (n = 22)
Age (years)	54.78 ± 16.20	62.27 ± 23.33
Male/female (n/n)	15/17	10/12
Risk stratification		
High risk (n)	1	/
Intermediate risk (n)	15	/
Low risk (n)	16	/
Main clinical symptoms		
Dyspnea	10	22*
Thoracic pain	11	18*
Clinical signs of DVT	15	0*
Risk factors		
Cancer	4	2
Recent surgery (< 30 days)	3	2
Previous DVT	9	0*
Previous PE	8	0*

*, P < 0.05. DVT, Deep vein thrombosis; PE, pulmonary embolism.

**Figure 3:**
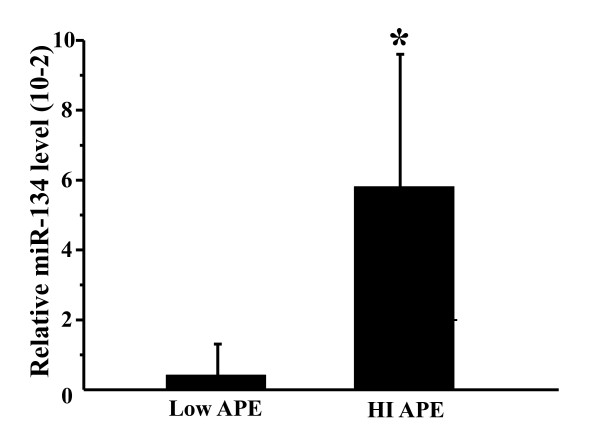
**Relative plasma microRNA-134 levels in low-risk acute pulmonary embolism and high-intermediate-risk acute pulmonary embolism**. APE, acute pulmonary embolism; Low APE, low-risk acute pulmonary embolism; HI-APE, high-intermediate-risk acute pulmonary embolism; miR-134, microRNA-134. The data are normalized by the expression levels of microRNA-16. *, P < 0.05, compared to high-intermediate-risk acute pulmonary embolism patients.

The plasma miR-134 level was increased in the APE group compared to both healthy controls and the non-APE group (Figure 1A). However, plasma miR-410 was also increased in the non-APE group (Figure 1B), indicating that miR-410, although initially identified by comparing the APE group to the healthy controls with miRNA microarrays, was also elevated in the non-APE group and therefore was not further studied. To confirm that the assay is reproducible, we also analyzed miR-134 expression level in the plasma (second sampling) collected 1 h after the first sampling of the plasma of three APE patients. No significant difference in the level of miR-134 was found between the first sampling and the second sampling, which indicates that the assay is reproducible (Additional file 2 Figure S2). We also compared the plasma miR-134 level between high-intermediate-risk APE and low-risk APE and found that the miR-134 level was significantly higher in the high-intermediate-risk APE patients compared to low-risk patients (Figure 3A).

### Diagnostic accuracy of plasma miR-134 for APE

**Figure 4:**
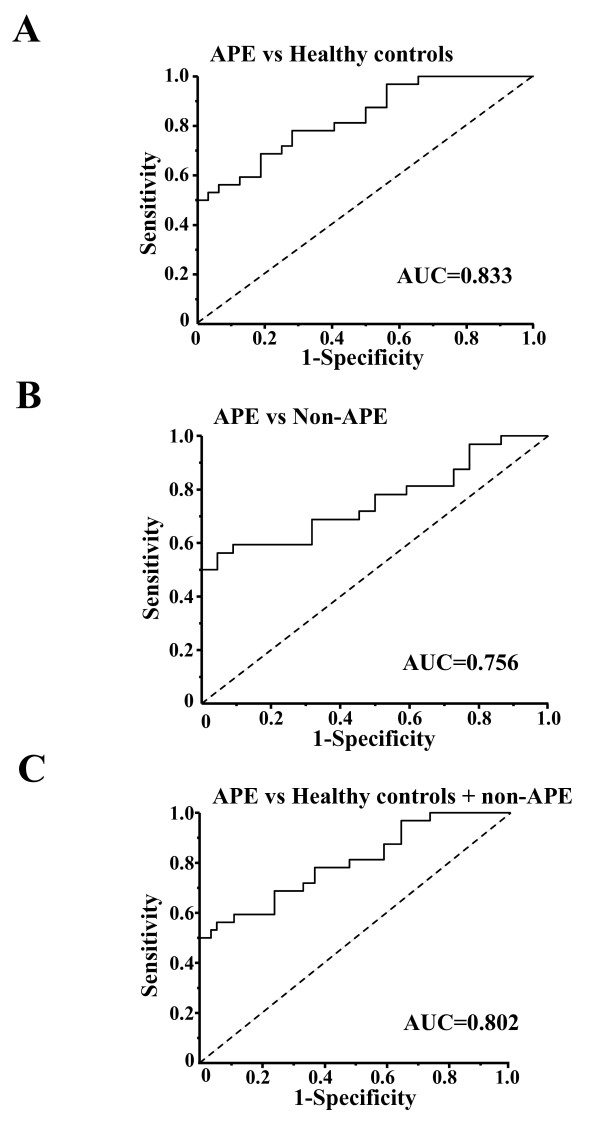
**The receiver-operator characteristic (ROC) curves for plasma miR-134 for acute pulmonary embolism (APE)**. (A-C) The receiver-operator characteristic (ROC) curves for distinguishing acute pulmonary embolism (APE) patients from healthy controls, non-APE patients and healthy controls plus non-APE patients, respectively. AUC, area under the ROC curve.

The ROC curve analysis was used to analyze the diagnostic accuracy of plasma miR-134. When a comparison was made between the APE group and the healthy controls, the AUC was 0.833 (95% confidence interval, 0.737 to 0.929; P < 0.001) (Figure 4A). When a comparison was made between the APE group and the non-APE group, the AUC was 0.756 (95% confidence interval, 0.629 to 0.883; P = 0.002). Using 0.00272375 as a cutoff value for the plasma miR-134 relative expression level, the sensitivity and specificity of miR-134 for the diagnosis of APE in patients reporting dyspnea, chest pain, or cough were 68.8% and 68.2%, respectively (Figure 4B). In addition, plasma miR-134 distinguished APE cases from healthy controls plus non-APE cases with an AUC of 0.802 (95% confidence interval, 0.702 to 0.901; P < 0.001) (Figure 4C).

## Discussion

APE has a nonspecific clinical presentation and is difficult to diagnose [24]. Although there has been great progress in the detection and exclusion of APE with the advent of D-dimer testing and chest CT scans, there is still a great need for simple and reliable biomarker detection for objective diagnostic testing of APE [7,31]. In this study, we have identified plasma miR-134 as a potential biomarker for APE.

Ideally, a biomarker should be reproducible and have a high sensitivity and specificity for the diagnosis of a specific disease [10]. miRNAs are exciting potential biomarkers because they fulfill many of these criteria [10,16]. Interestingly, miRNAs are present in human plasma in a remarkably stable form that is protected from endogenous RNase activity and remain stable even after being subjected to harsh conditions [15,32]. The stability, low structural complexity, and lack of post-processing modifications make plasma miRNAs ideal biomarker candidates [16]. The high sensitivity and specificity of miRNA detection using qRT-PCR may create precise cutoff concentrations for diagnosis [15,17]. To date, the role of plasma miRNAs in APE has not been reported. As demonstrated by the miR-134 identified in this study, the use of miRNAs as minimally invasive and robust biomarkers would result in incredible breakthroughs for the diagnosis of this common disease.

Spiral CT arteriography is widely used in the clinic. The sensitivity of spiral CT arteriography alone was 83%, whereas the combination of CT arteriography and CT venography increased the sensitivity to 90%. However, in those patients with unavailable multidetector CT, renal failure or an allergy to contrast dye, plasma miR-134 might be an ideal alternative. In addition, using plasma miR-134 detection reduces the overall radiation exposure compared to CT [1,4,24].

We have demonstrated that the plasma miR-134 level was not affected by non-APE conditions. Plasma miR-134 could distinguish APE cases from healthy controls or non-APE cases with an AUC of 0.833 or 0.756, respectively, which indicates the potential for using plasma miR-134 as a biomarker to diagnose APE. Interestingly, a recent report indicated that the expression of miR-134 was 3.5-fold higher in the peripheral blood mononuclear cells of unstable angina pectoris patients compared to those with stable angina pectoris [33]. In this study, the plasma miR-134 level in 7 patients with unstable angina pectoris and 3 with an acute myocardial infarction was similar to healthy controls. This discrepancy may have been due to the difference between miRNA levels in plasma versus peripheral mononuclear cells used in the original report [33]. In addition, Wong et al. found an increased expression of miR-134 in patients with squamous cell carcinoma. However, their study used tissue, whereas our study used plasma [26].

Our study was subject to a number of limitations. First, the number of APE patients in this study is relatively small. The results obtained in this small group will require replication in large, independent studies of APE. Second, it would be interesting to analyze the relationship of miR-134 to the laboratory findings in both APE and non-APE patients. For example, it would be helpful to investigate whether combining the values of D-dimer and miR-134 would greatly enhance the specificity and sensitivity for plasma miR-134. Some non-APE patients in this study did not have D-dimer results because all of the collected plasma was used to isolate total RNA. Further studies are needed to resolve this issue. However, the D-dimer test has a high sensitivity but is non-specific because it is also positive in patients with infection, cancer, and other inflammatory diseases [1,24]. The specificity of miR-134 in diagnosing APE was better than D-dimer test in this study. Third, further work is required to determine the additive benefit of miR-134 in algorithms for APE detection that incorporate other diagnostic modalities in prospective fashion. Fourth, whether circulating miRNAs have a biological function is unclear. It is commonly speculated that circulating miRs play a role in maintaining the homeostasis of the circulatory system [30]. Whether plasma miR-134 can trigger some pathogenic or protective effects in APE remains uncertain. Finally, the pathobiological mechanism of miR-134 levels and the relationship with APE is unclear. Prior studies have demonstrated that the release of miRNA from exosomes, microparticles, or apoptotic bodies may be the cause of miRNA dysregulation in disease [24,34].

## Conclusions

In conclusion, we have found that elevated plasma miR-134 levels are a potential novel biomarker for the diagnosis of APE. Our results provide a basis for future efforts to develop plasma miRNA-based assays to diagnose APE.

## Competing interests

The authors declare that they have no competing interests.

## Authors' contributions

JJX and DDL conducted the TaqMan microRNA array; ZCJ, XLC and LP enrolled patients and recorded clinical data; HZ, YL, and YL performed the TaqMan-based miRNA quantitative real-time reverse transcription polymerase chain reactions; LYP, XQL, and YFS performed the statistical analysis and reviewed the manuscript; PTE, RL, LMP and GC analyzed the data and reviewed the manuscript. YHC designed the study and wrote the manuscript. All authors read and approved the final manuscript.

## Supplementary Material

Additional file 1 **Figure S1 - Relative plasma miR-16 levels in acute pulmonary embolism patients**. APE, acute pulmonary embolism. Click here for file

Additional file 2 **Figure S2 - Relative plasma microRNA-134 levels in the first sampling and second sampling**. n.s., not significant. Click here for file
