# Supplementary figures and images for: MicroRNA-134 as a potential plasma biomarker for the diagnosis of acute pulmonary embolism
Source: J Transl Med. 2011 Sep 24;9:159. doi: 10.1186/1479-5876-9-159 (PMC3189884; doi:10.1186/1479-5876-9-159)

## Slide 1
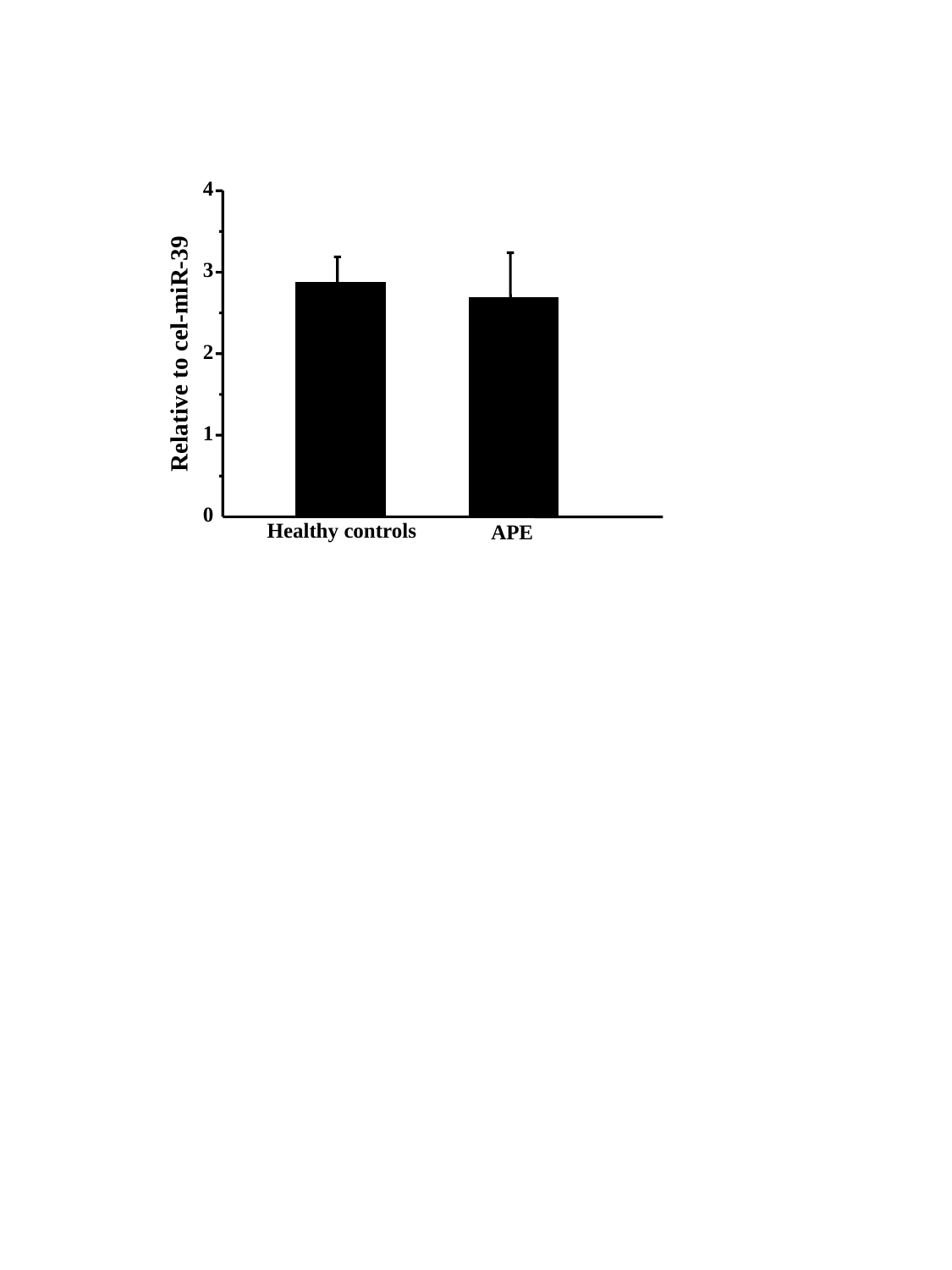

4
3
2
1
0
Relative to cel-miR-39
Healthy controls
APE

Supplement: Additional file 1 — Figure S1 - Relative plasma miR-16 levels in acute pulmonary embolism patients. APE, acute pulmonary embolism. [file 1479-5876-9-159-S1.PPT]

## Slide 1
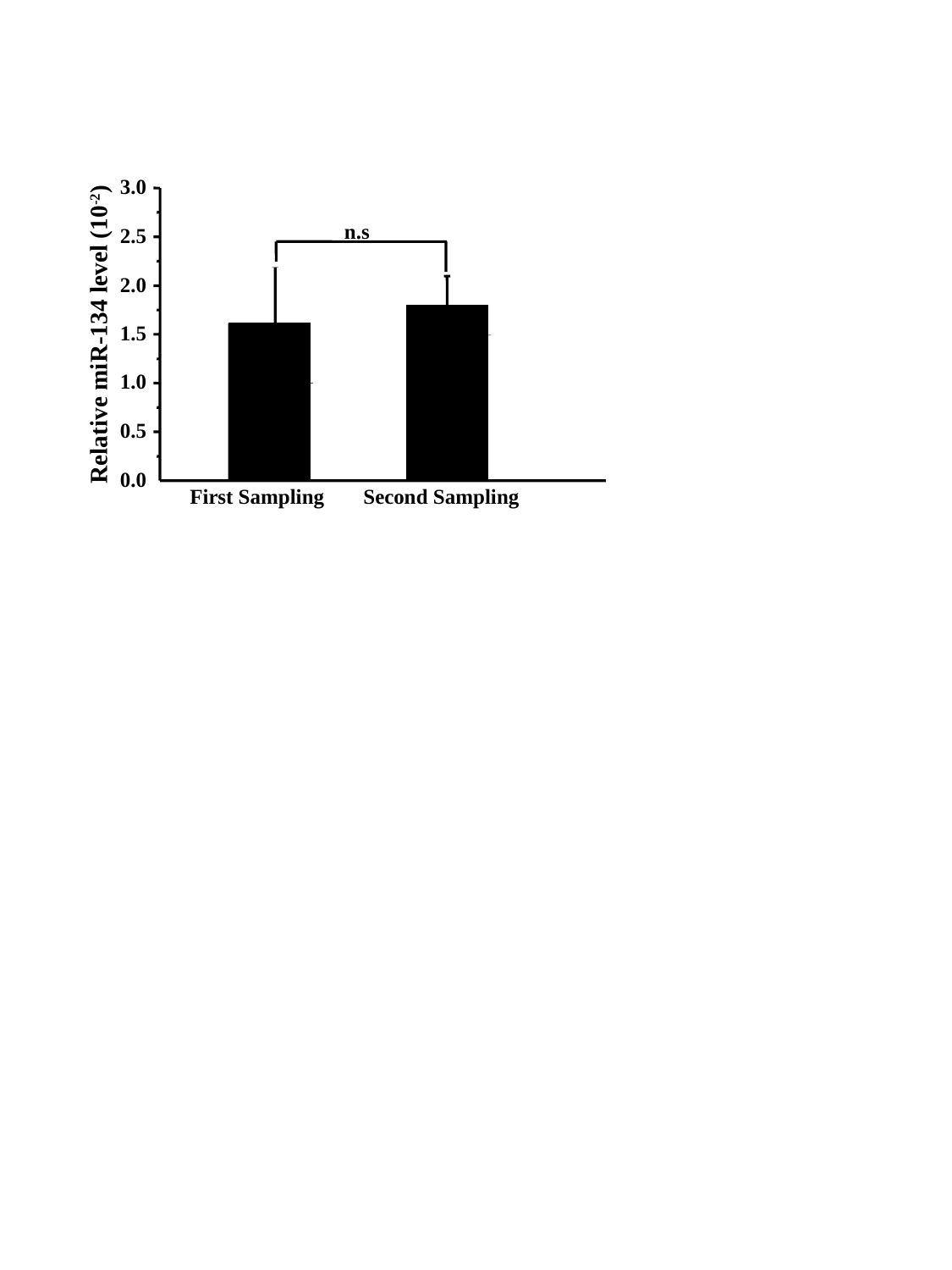

3.0
2.5
2.0
1.5
1.0
0.5
0.0
n.s
Relative miR-134 level (10-2)
Second Sampling
First Sampling

Supplement: Additional file 2 — Figure S2 - Relative plasma microRNA-134 levels in the first sampling and second sampling. n.s., not significant. [file 1479-5876-9-159-S2.PPT]
